# Validation of the Early Warning and Response System (EWARS) for dengue outbreaks: Evidence from the national vector control program in Mexico

**DOI:** 10.1371/journal.pntd.0009261

**Published:** 2021-12-16

**Authors:** David Benitez-Valladares, Axel Kroeger, Gustavo Sánchez Tejeda, Laith Hussain-Alkhateeb

**Affiliations:** 1 Programa de Enfermedades Transmitidas por Vector, Centro Nacional de Programas Preventivos y Control de Enfermedades, CENAPRECE, Secretaría de Salud de México, Ciudad de México, México; 2 Special Programme for Research and Training in Tropical Diseases (TDR) at the World Health Organization in Geneva, Geneva, Switzerland; 3 Albert-Ludwigs-University Freiburg, Master Programme Global Urban Health, Freiburg, Germany; 4 Global Health, School of Public Health and Community Medicine, Sahlgrenska Academy, University of Gothenburg, Sweden; Australian Red Cross Lifelood, AUSTRALIA

## Abstract

**Background:**

During 2017, twenty health districts (locations) implemented a dengue outbreak Early Warning and Response System (EWARS) in Mexico, which processes epidemiological, meteorological and entomological alarm indicators to predict dengue outbreaks and triggers early response activities.

Out of the 20 priority districts where more than one fifth of all national disease transmission in Mexico occur, eleven districts were purposely selected and analyzed. Nine districts presented outbreak alarms by EWARS but without subsequent outbreaks (“non-outbreak districts”) and two presented alarms with subsequent dengue outbreaks (“outbreak districts”). This evaluation study assesses and compares the impact of alarm-informed response activities and the consequences of failing a timely and adequate response across the outbreak groups.

**Methods:**

Five indicators of dengue outbreak response (larval control, entomological studies with water container interventions, focal spraying and indoor residual spraying) were quantitatively analyzed across two groups (”outbreak districts” and “non-outbreak districts”). However, for quality control purposes, only qualitative concluding remarks were derived from the fifth response indicator (fogging).

**Results:**

The average coverage of vector control responses was significantly higher in non-outbreak districts and across all four indicators. In the “outbreak districts” the response activities started late and were of much lower intensity compared to “non-outbreak districts”. Vector control teams at districts-level demonstrated diverse levels of compliance with local guidelines for ‘initial’, ‘early’ and ‘late’ responses to outbreak alarms, which could potentially explain the different outcomes observed following the outbreak alarms.

**Conclusion:**

Failing timely and adequate response of alarm signals generated by EWARS showed to negatively impact the disease outbreak control process. On the other hand, districts with adequate and timely response guided by alarm signals demonstrated successful records of outbreak prevention. This study presents important operational scenarios when failing or successding EWARS but warrants investigating the effectiveness and cost-effectiveness of EWARS using a more robust designs.

## Author summary

While the Early Warning and Response System (EWARS) for dengue and other vector-borne diseases has significantly advanced in methodologies and applications in more recent time, evidence from the field on the implications of EWARS in the context of vector control and response is still rare. Assessing the effectiveness (and cost) of EWARS in reducing or mitigating disease outbreaks entails complex study designs, including randomized controlled trials. This study employs a pragmatic validation design; comparing groups of outbreak and non-outbreak districts from the Mexican national vector control and response program for dengue, all districts with implemented EWARS and generated alerts. The study authors have illustrated and discussed important operational implications when failing or succeeding timely and adequate response to EWARS alerts, revealing that responding to EWARS alerts in time and space, during or beyond the routine vector control and response process can significantly reduce unnecessary outbreaks and hospitalizations.

## Introduction

Dengue, a mosquito-borne viral disease, is currently one of the most important and fastest-spreading infectious diseases in the world, putting a significant burden on populations, health systems and economies in most tropical and sub-tropical countries [1]. There are four viral serotypes circulating in Asia, the Americas and Africa, with an estimated 3.6 billion people living in dengue endemic countries, and about 300 million infections occuring annually [2]. The growing global threat of dengue outbreaks in endemic and non-endemic regions, steers the public health communities towards efficient disease outbreak control.

Routine passive surveillance systems for dengue and other infectious diseases are the prime source of epidemiological information in the vast majority of countries, which facilitate the analysis of the spatial and temporal distribution of clinically apparent dengue cases. Such data can highlight "hot spots" and priority areas for interventions and serve as alarms for local implementation of prevention and control activities [3]. However, the underreporting of cases, especially of non-hospitalized dengue cases (in addition to asymptomatic or mild disease, non-users of the public health sector and others) is common and can have vector control implications. National surveillance systems have to use appropriate tools to trigger response actions for outbreaks and these must be sensitive to detect unusual trend and potentially predict outbreaks in a timely manner and with least false outbreak alerts.

The early warning of outbreaks and the subsequent response is crucial to reduce human suffering and economic losses, both for families affected by the disease, and for health systems that are already weakened in most dengue endemic countries [4]. By virtue of the lack of effective vaccine, the early warning system will be best alternative, which must typically be followed by a rapid outbreak response guided by standard operational procedures (SOPs).

The Special Programme for Research and Training in Tropical Diseases (WHO/TDR) initiated together with European research institutions, the national dengue control services and academia in endemic partner countries, including the centre for vector control at the Ministry of Health in Mexico (CENAPRECE), the development of a web-based Early Warning And Response System (EWARS) for dengue outbreaks [4–6]. Throughout multi-country qualitative and quantitative assessments, the tool has revealed consistent outbreak predictions with instant interpretations including a subsequent action plan using the local vector control response protocol. The tool can be easily integrated into existing surveillance systems, which maintained high acceptability by users, and managed to strengthen the communication and collaboration between the central (national) and district levels, and promoted national and international partnerships [7]. The TDR-EWARS has more recently been evaluated for chikungunya, Zika and malaria outbreaks and the study findings are at the dissemination phase.

In the context of EWARS, interpreting essential terms such as alarm signals (notifications generated by the prediction model when the computed probability of an upcoming outbreak exceeds the defined outbreak threshold in a particular district), “correct alarms” (alarm signals that were followed by an outbreak or would have been followed by an outbreak,if no interventions would have taken place) and “false alarms” (alarm signals that were -without interventions- not followed by an outbreak) have significant local operational implications for a functioning surveillance system. Factors related to the timeliness and intensity of response activities are crucial for mitigating or averting the outbreak, and these factors can potentially define the effectiveness of the EWARS in reducing unwanted disease outcomes in a particular area. The association between response activities (timeless and intensity) and the benefits of alarm signals generated by EWARS is rarely explored in the context of vector-borne diseases. In order to ensure effective response, EWARS should be perceived as an information system designed to support decision making at national and local-levels. It should be able to improve coordination among relevant stakeholders, including epidemiologists, meteorologists and the public communication channels used to disseminate warning information as well as the national and local management agencies responsible for assessing risk and develop response strategies.

During 2017, there were around 90,000 probable cases and 14,000 confirmed cases in Mexico. The geographical distribution of dengue in Mexico covers almost all national states with more than half of all transmission (58% of probable cases and 53% of confirmed cases) is concentrated in 168 districts according. These are defined by the Mexican Secretary of Health as priority localities for dengue [8]. However, 20 districts out of the 168 areas define the priority “hot spot” of dengue transmission where more than one fifth of all national tranismission is reported. With this in view, the aim of this study was to examine and compare outbreak response activities and their impact on the outbreak prevention in districts with alarms followed by outbreaks (“outbreak districts with alarm signal”), and in districts which did not report outbreaks after the alarm (“non-outbreak districts with alarm signals”). We therefore hypothesized that i) delayed response activities measured by the timing (initial, early or late response) and ii) low intensity of the response in terms of low coverage or frequency of interventions are associated with outbreaks after receiving alarm signals.

## Methods

### Ethics statement

Ethical approval was obtained by the Pan American Health Organization Ethics Review Committee (PAHO ERC 2011-12-021). No specific permits or informed consents were required for the described field studies; the locations are not privately-owned and the field studies did not involve endangered or protected species.

The prospective implementation of the EWARS tool in Mexico was carried out during 2015–2017, but this evaluation study will focus on 2017 when the implementation was completed with the highest degree of quality due to capacity building of surveillance staff in the study districts.

Of the priority 20 districts that participated in the implementation of EWARS, only eleven presented outbreak alarms, which defined our study sample. During the 2017 study, two out of eleven districts reported outbreaks after the alarm and nine did not show outbreaks, taking into account the WHO and UNICEF operational definition of an outbreak [6]. The “outbreak districts with alarm signals” were; *Ciudad Apodaca* and *San Nicolás de los Garza* (both in the State of Nuevo León). “Non-outbreak districts with alarm signals” were; *Culiacán* and *Mazatlán* (both in the State of Sinaloa), metropolitan area of ​​*Veracruz* and Coatzacoalcos (both in the State of Veracruz), *Apaztingan* and *Zamora* (State of Michoacán), *Cardenas* (State of Tabasco), *Iguala* (State of Guerrero) and, *Monterrey* (State of Nuevo Leon).

As a conceptual design, the EWARS employs the endemic channel using area-specific disease data (i.e. district, province or locality level data)–the endemic channel represents a smoothed weekly moving mean and standard deviation, based on data in the historic period. Using a multiplier of the standard deviation known as ‘z’, it was possible to vary the endemic channel within the evaluation period. The in-control state of disease is when the number of cases remains within the expected normal range, while anything above this moving average is considered representative of an unusual number of cases (i.e., an outbreak). EWARS does not merely rely on outbreak records (i.e. outbreak detection) but utilizes a broad spectrum of epidemiological, entomological and meteorological data (alarm indicators) to inform about a forthcoming dengue outbreak, applying the logistic and Poisson regression modelling (i.e. prediction). Applying structured calibration steps, users are able to define relevant measures such as the size of the endemic channel, the outbreak duration, the alarm threshold and the prediction distance and test different threshold points, which can optimize the sensitivity (percent of alarms generated by the tool in relation to the outbreaks observed in the locality) and positive predictive value of the prediction model (the percentage of correct alarms in relation to all generated alarms) [5,7].

In order to analyze the staged outbreak response (‘initial’: *is declared when two consecutive alarm signals occur*; ‘early’: *is declared when three consecutive alarm signals occur*; and ‘late’ response: *is declared when more than three consecutive outbreak weeks take place*, see Table 1), data of all dengue control activities during 2017 were obtained from the study districts using the online entomological surveillance platform of Mexico.

**Table 1:** Type of response and the corresponding dengue control activities.

Type of response	Initial response	Early response	Emergency or late response
Dengue control activities	Communicating risk to authorities, local dengue committees and outbreak management team at hospitals; Updating the necessary background information such as cartography, demographics and inventory of facilities; Focused vector control in areas considered historically high transmission.	Communicating increased outbreak risk to authorities, local dengue committees and outbreak management team at hospitals; Convening local dengue or emergency operations committees; Social mobilization (IEC, community participation, mass media partnerships); Enhance communication channels with Public Health, Clinical, Education System., Media, national and international authorities; Vector control target the adult mosquito stages (focused around cases) with limited spatial fogging	Full application of the contingency plans.

The Mexican entomological surveillance platform divides response activities into five components: larval control (using different types of larvicides as well as physical control by removing breeding sites [9]), entomological studies (to obtain vector indexes, mainly House Index for *Aedes aegypti*, to assess the quality of the interventions done and to treat or remove additional breeding sites), focal spraying (i.e. indoor space spraying of all houses of the block where the house of the case is located [9]), indoor space spraying and, outdoor fogging (space spraying with Hudson pumps [9]).

To serve the purpose of this study, an indicator has been created for each response:

1. Larval control indicator: Number of houses covered per epidemiological week.
2. Indicator of entomological studies (which includes vector control activities): Number of entomological studies per epidemiological week.
3. Residual spray indicator around probable cases (perifocal spraying): Number of probable cases where perifocal spray activities was conducted per epidemiological week.
4. Indoor residual spray indicator: number of houses covered by indoor space spraying per epidemiological week.
5. Fogging (space spraying) indicator: area covered (Km2) per epidemiological week

The numbers provided by the Mexican national surveillance platform were divided by the estimated number of houses or households per district to get weekly “proportion of houses served”. Nevertheless, these are not coverage rates per se (as coverage would refer to “number of houses served divided by total number of target houses”). Since the response activities are focusing on high risk areas only (i.e. transmission hot spots) and the target houses are not published, we used the estimated number of houses per district as the denominator. Most families in the study districts live in separated houses while there were also multistory buildings with separate apartments (“households”), therefore we use the term “house” and “household” interchangeably.

### Data management and statistical analysis

Due to inconsistencies in measuring the “*fogging (space spraying)*” during the data collection process, we only analyzed and discussed the other four response indicators quantitatively with some concluding remarks about the realities of space spraying (fogging). The intensity of vector control activities (i.e. % of houses covered) at each district was calculated before and after the alarm signal period (week) for each of the four response indicators. The proportion of houses served was calculated by dividing the total number of covered households, by the total number of households in the corresponding district for the *larval control*, *indoor space spraying* and *entomological studies*. For the *spraying of houses around new cases* (“perifocal spraying”), this indicator was computed by dividing the number of perifocal spraying activities by the total number of houses in the corresponding district.

Our study hypothesis of the association between delayed or low-intensity response activities after outbreak alarms and dengue outbreaks was tested by employing mix-effect linear regression and Generalized Estimating Equations (GEE) models to account for the data assumptions and the repeated measure effects. Segmented time-series analysis was used to examine the trend of intensity of vector control activities in each district and at three different occasions; 1) at baseline (the period prior to the EWARS alarm signal), 2) at the time point (week) of the alarm signal (this measures the immediate change in vector response between the baseline period and one week after the alarm signal) and, 3) at the remaining period (25 weeks) following the alarm signal for each district. These three different trends were presented as “rates of change in percentage or per 1000 population” together with their p-values at 5% significant cut off. Due to the internal procedures of the independent Mexican epidemiological and entomological surveillance systems, there is a one week time lag between obtaining the surveillance data and processing the prediction model of the EWARS tool. Furthermore, dengue control activities are usually planned on a weekly basis, which defines the alarm-response operational period. The time lag between an alarm and a response is usually two weeks; the prospective collection of alarm indicators information, their entry into the EWARS to generate outbreak prediction and, the weekly schedule of control activities were all taken into account when planning this analysis. This time lag mechanism defined the ‘alarm period’, which is set in our study at epidemiological week 28 for the segmented time-series analysis. Since the process of vector control and response activities are standardized at the national level, we applied the same epidemiological week (week 28) for all analyzed districts. Descriptive statistics, in numbers and graphs, were also produced and presented before and after the alarm signal week, generated from the EWARS, for the corresponding district-groups and indicators.

**Fig 1:**
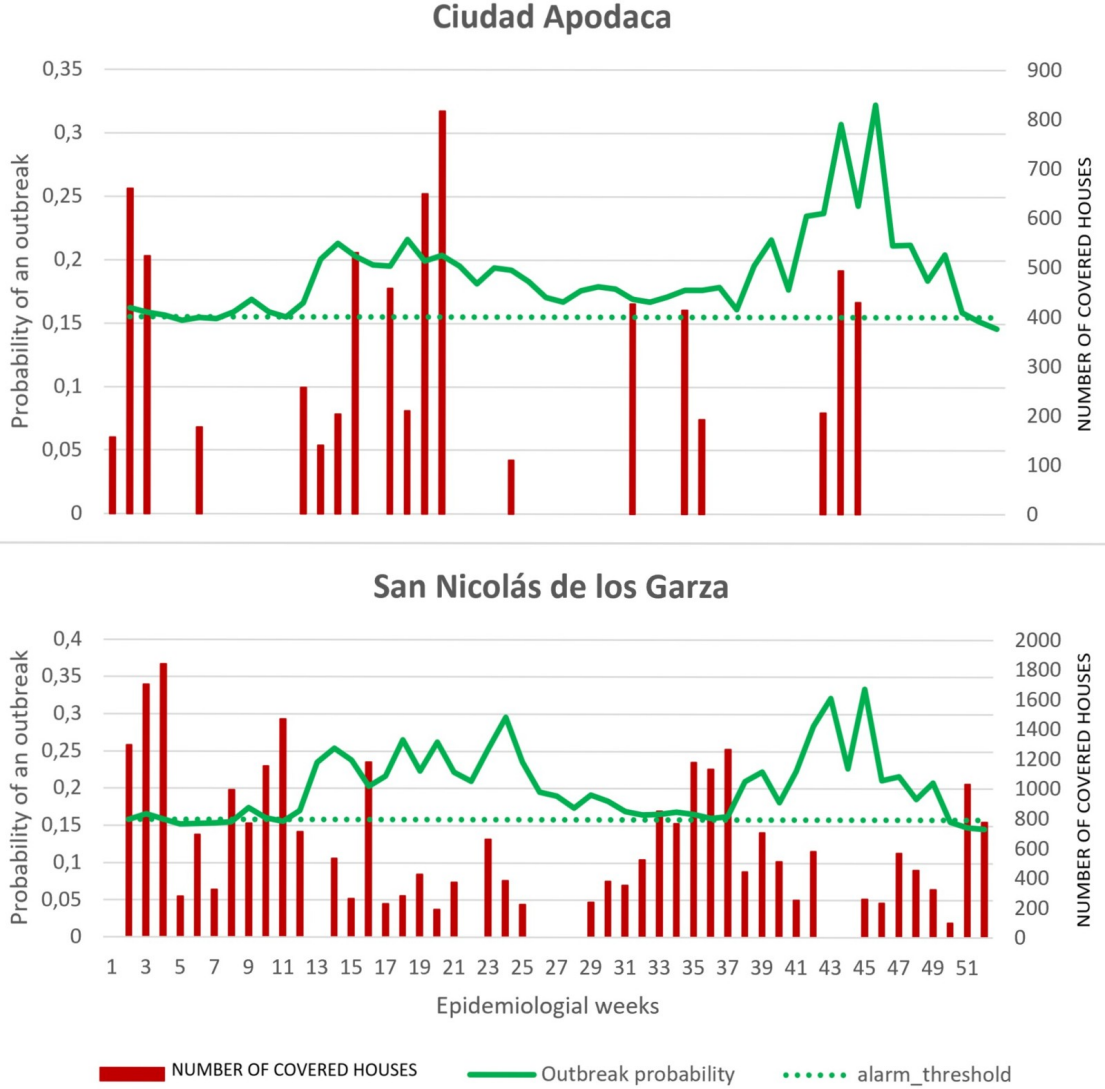
Time-series of weekly number of houses covered with larval control against the probability of outbreaks at before and after the alarm signals (week 28), in outbreak districts.

**Fig 2:**
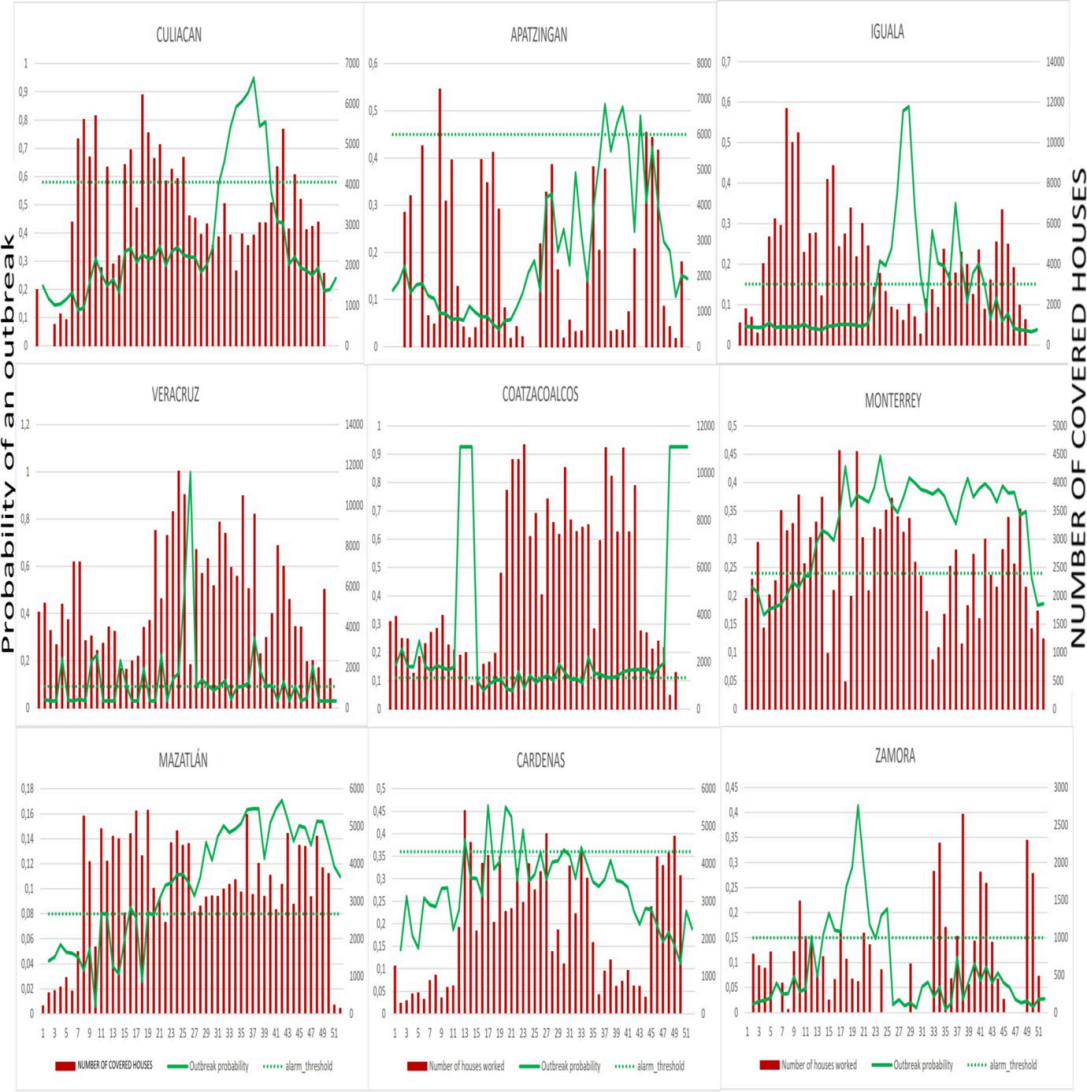
Time-series of weekly number of houses covered with larval control against the probability of outbreaks at before and after the alarm signals (week 28), in non-outbreak districts.

**Fig 3:**
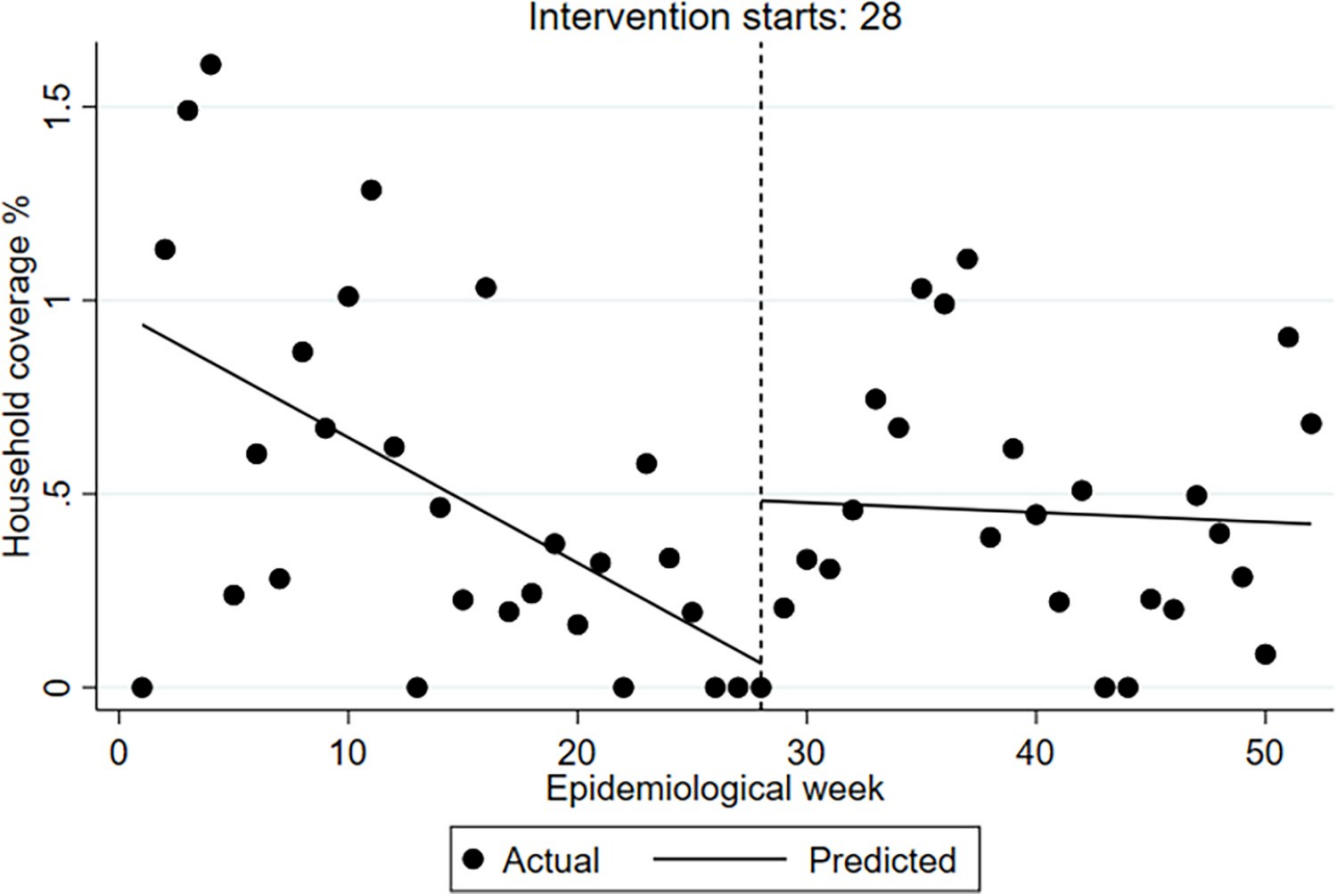
Segmented time-series of household coverage % with larval control at baseline (pre-alarm signal period), at the week (28) of alarm signal and post-alarm signal period. One example of outbreak districts is presented. (San Nicolas).

**Fig 4:**
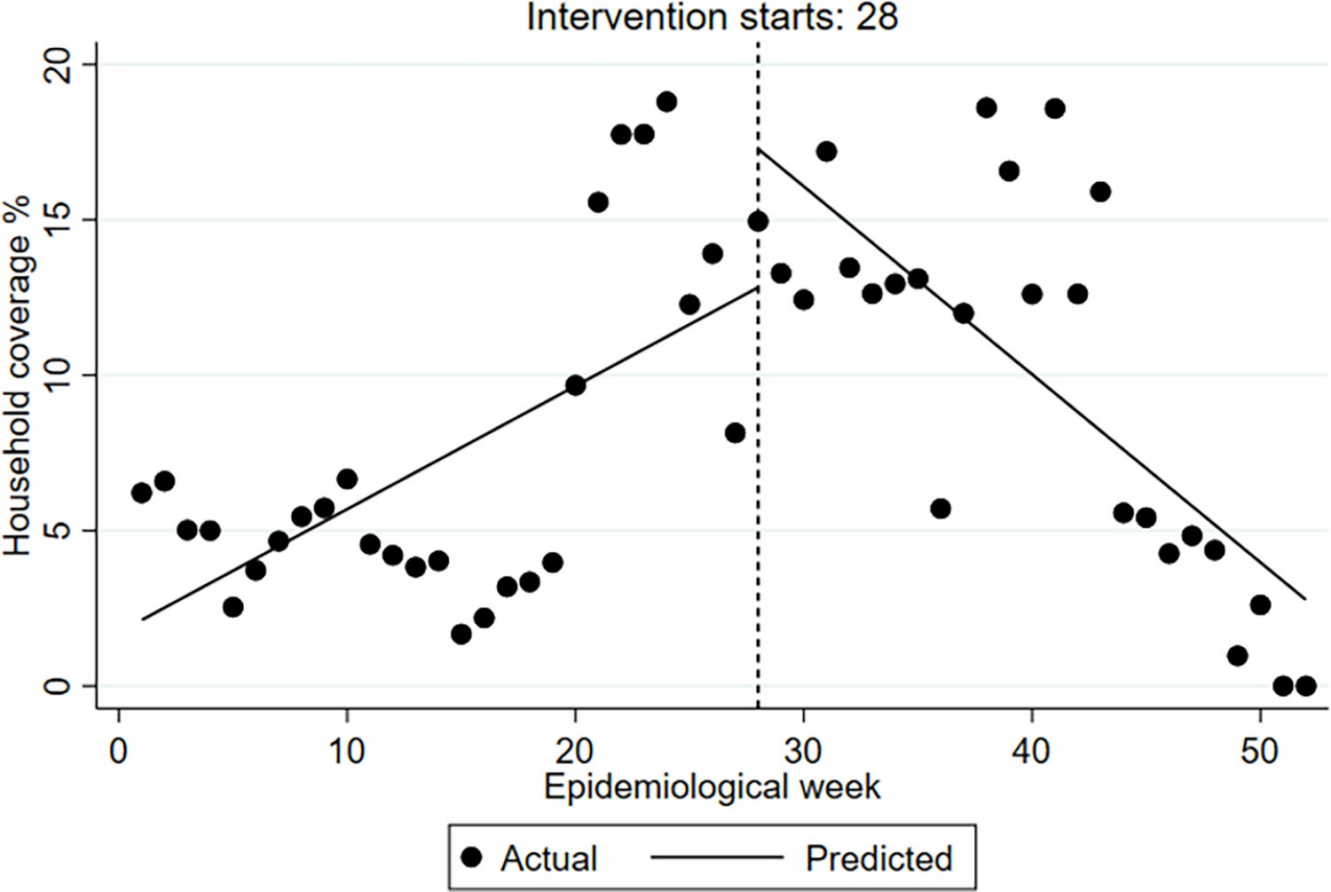
Segmented time-series of household coverage % with larval control at baseline (pre-alarm signal period), at the week (28) of alarm signal and post-alarm signal period. One example of non-outbreak districts is presented. (Coatzacoalcos).

We took the larval control as an example from the four indicators to illustrate the vector control activities across the outbreak and non-outbreak district groups, showing activities at the pre- and post-alarm period (week 28 is the alarm week). Figs 1 and 2 (outbreak districts with alarm signals group, and non-outbreak districts with alarm signals group) show the weekly number of households covered at pre- and post-alarm period and provides a more aggregated presentation of vector control activities at pre- and post-alarm periods. Figs 3 and 4 (one outbreak district with alarm signals, and one non-outbreak district with alarm signals) show the trends of activities before and after the alarm signal turning positive.

## Results

**Fig 5:**
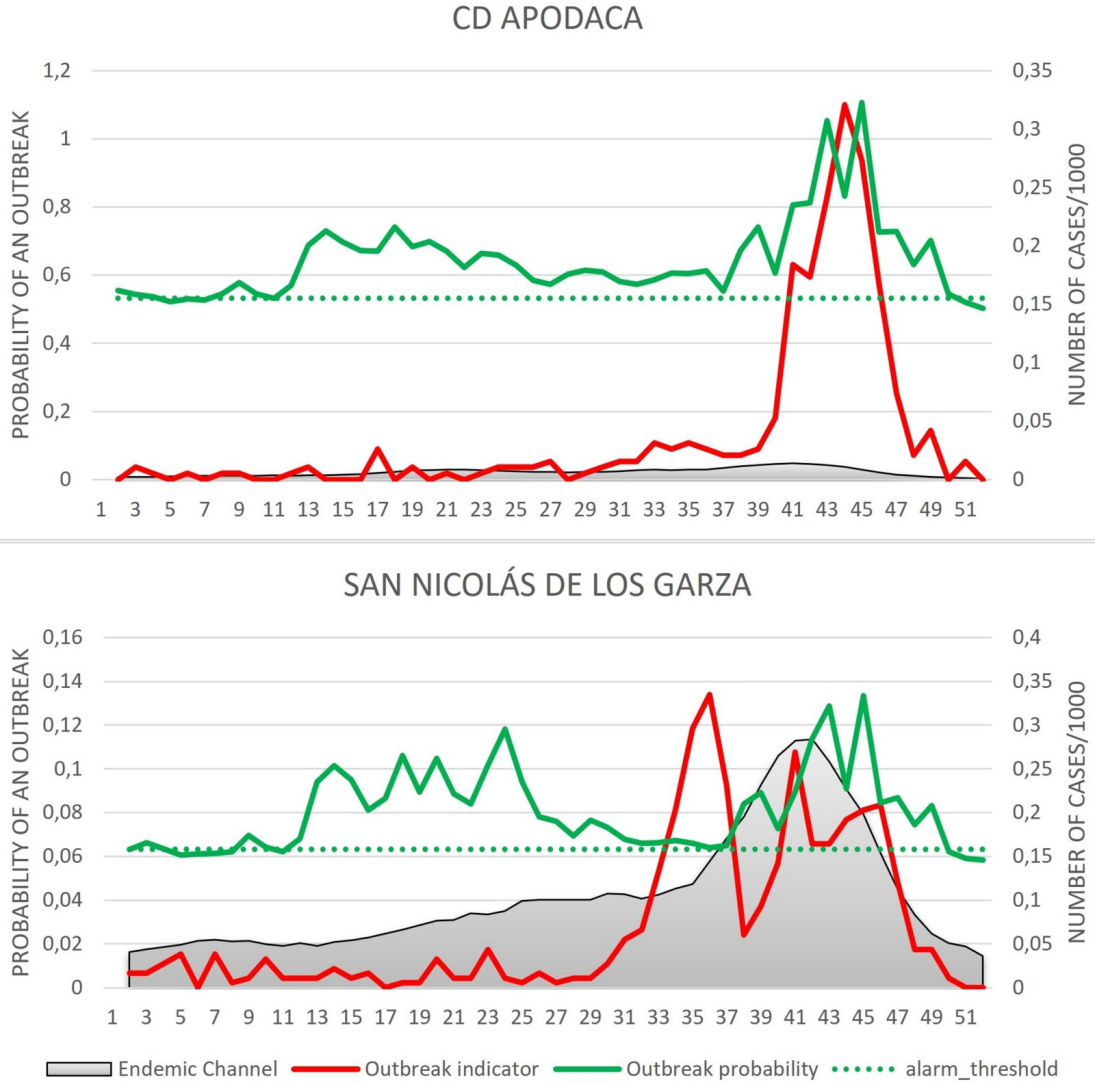
The outbreak predictions as generated by the EWARS for the outbreak districts. The endemic channel is presented by the shaded area in grey which includes + z* SD of the moving average; the alarm threshold is represented by the dotted horizontal line; the outbreak probability (“alarm line”) estimated by the EWS is represented by the green line; the weekly dengue incidence rates is represented by the red line.

**Fig 6:**
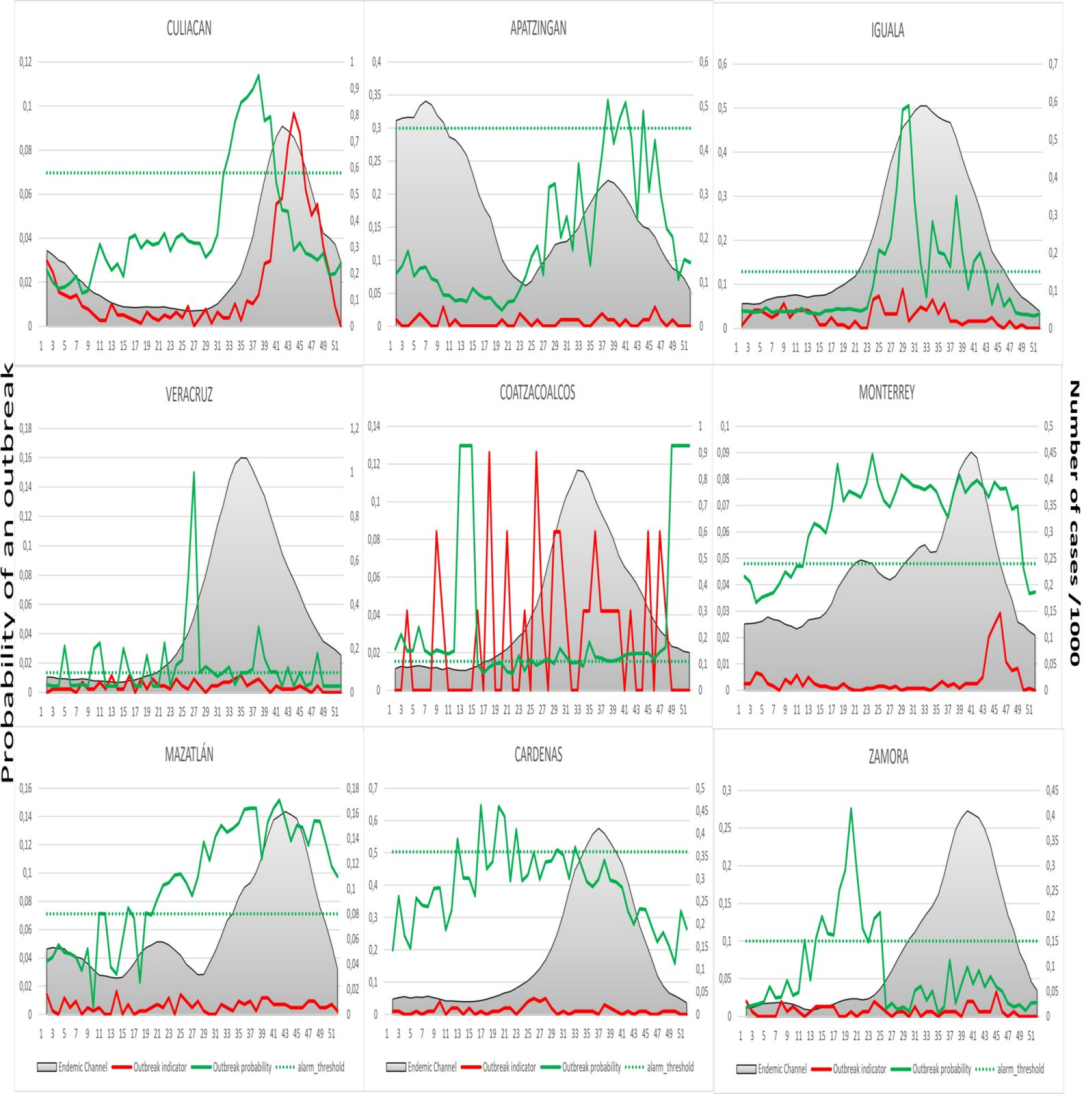
The outbreak predictions as generated by the EWARS for the non-outbreak districts. The endemic channel is presented by the shaded area in grey which includes + z* SD of the moving average; the alarm threshold is represented by the dotted horizontal line; the outbreak probability (“alarm line”) estimated by the EWS is represented by the green line; the weekly dengue incidence rates is represented by the red line.

Figs 5 and 6 demonstrate the outbreak prediction scenario of the corresponding district, with Fig 5 representing the “outbreak districts with alarm signals group” and Fig 6 representing the “non-outbreak districts with alarm signals group”. The endemic channel for each epidemiological week during 2017 with the upper line being the outbreak threshold is illustrated together with the alarm threshold and the estimated outbreak probability by the EWARS using climatic, epidemiological and entomological indicators. Alarm signals are triggered once the outbreak probability (“alarm line”) exceeds the alarm threshold line indicating a probable outbreak. The weekly dengue incidence rates are also shown to inform about the prospective distribution of cases in relation to historical incidences (endemic channel). In “outbreak districts with alarm signals”, the alarm signal is followed by an outbreak, whereas in the “non-outbreak districts with alarm signals”, the alarm signal is not followed by an outbreak.

### Vector control indicators in outbreak and non-outbreak districts

Table 2 summarizes the results of the association between the four control indicators and the outbreak/non-outbreak groups. In general, vector control activities were significantly lower among the outbreak districts with alarm signals group compared to the non-outbreak districts with alarm signals group, with the exception of the *intensity of indoor spraying* indicator, which showed a non-significant association between both outbreak and non-outbreak groups, althoght the intensity of idoor spraying was higher in the “non-outbreak group”. Outbreak districts with alarm signals showed reduced larval control activities and to a less extent, reduced entomological studies and focal spraying (i.e. spraying around cases).

**Table 2:** Mix-effect linear regression and GEE of the association between the intensity of different types of vector control and outbreak/non-outbreak groups.

Type of control indicator	Outbreak Group	Coef. (95% CI)	P-value
Intensity of larval control	Non-outbreak districts Outbreak districts	Ref. -1.98 (-3.21 to -0.75)	0.002
Intensity of Entomological studies	Non-outbreak districts Outbreak districts	Ref. -1.54 (-2.40 to -0.70)	0.001
Intensity of Spraying around cases	Non-outbreak districts Outbreak districts	Ref. -0.12 (-0.22 to -0.03)	0.012
Intensity of indoor spraying control	Non-outbreak districts Outbreak districts	Ref. -1.47 (-3.99 to 1.05)	0.254

### Time series analysis of rate of change of vector control indicators in outbreak and non-outbreak districts

#### Larval control indicator

Different types of larvicidal and physical control are used to treat or remove breeding sites, including pyriproxyfen and larvicides [9]. Table 3 presents the findings from the segmented time-series analysis and the percentage of household coverage at district-level for the larval control (see comments on coverage estimates in the introduction).

**Table 3:** Intensity of larval control. Percent of households before and after alarm signals and rate of change between periods in 2 outbreak and 9 non-outbreak districts with alarm signals.

	Intensity of Larval control (% of households reached out of all district households)		Rate of change in % of households reached (out of all houses at district-level), *Coef β (p-value)*		
	Before alarm signal	After alarm signal	Pre-alarm period (baseline)	Alarm period	Post-alarm period
**Outbreak district**	0.32	0.26	-	-	-
CD APODACA	0.13	0.06	-0.002	-0.008	0.001
SAN NICOLAS	0.51	0.45	-0.032*	0.420*	0.029*
**Non-outbreak district**	4.93	4.48	-	-	-
VERACRUZ	4.54	5.01	0.115	1.785	-0.360*
MAZATLAN	3.04	3.16	0.130*	-1.390*	-0.157*
CULIACAN	1.76	1.46	0.075*	-1.125*	-0.094*
APATZINGAN	8.59	8.64	-0.087	2.541	-0.018
COATZACOALCOS	7.27	10.02	0.396*	4.457	-1.001*
CARDENAS	9.52	8.44	0.641*	-10.139*	-0.636*
IGUALA	16.49	9.13	0.038	-9.013*	0.054
MONTERREY	0.93	0.74	0.011*	-0.329*	-0.013
ZAMORA	1.41	2.22	-0.025	1.026	0.036

* Significant (p-value<0.01)

The proportion of households reached with the intervention was clearly much higher in the non-outbreak districts with alarm signals group compared to outbreak districts with alarm signals group, but it also varied considerably between districts with the highest proportion amongst the non-outbreak districts with alarm signals. From the segmented analysis, it is apparent that the rate of larval control was consistently declining among the outbreak districts with alarm signals and only slightly increased following the alarm signal (alarm period) generated by the early warning model. The non-outbreak districts with alarm signals group, however, maintained a routine increase of larval control rate at baseline (i.e. before any alarm occurred) with the exception of two districts out of nine (Apatzingan and Zamora). In contrast, the outbreak districts with alarm signals group attempted to slightly increase the rate of larval control only after the alarm signal (alarm period) turned positive but particularly after the case numbers increased. Most of the non-outbreak districts with alarm signals tended not to intensify or even reduce their house-to-house larval control, activities putting more emphasis on other potentially more efficient outbreak response activities (see Table 3).

### Entomological studies

In Mexico, vector control personnel perform entomological studies on a sample basis to control the quality of larval control activities and at the same time enhance larval control. Entomological studies are carried out to confirm vector indexes (such as House Index for *Aedes aegypti*), and to measure the quality of the interventions. During these studies, if a breeding site is discovered, vector control staff will mechanically remove or treat it with a larvicide or larval growth inhibitor (pyriproxyfen), thus the entomological studies include quality assurance and vector control activities.

According to the segmented time series analysis, the intensity of entomological studies followed a fairly similar pattern compared to that observed for the larval control indicator. In general, the proportion of houses reached by entomological studies was lower as these were being performed on a sample basis. However, the non-outbreak districts with alarm signals groups maintained a higher percentage of entomological studies compared to the outbreak districts with alarm signals group. Unlike the outbreak districts with alarm signals, the non-outbreak districts with alarm signals group showed an increased rate of activities at baseline (pre-alarm period), i.e. prior to any alarm signal, with exception of two districts. The rate of entomological studies varied across the groups at the post alarm period but remained at a low rate with the exception of the non-outbreak districts Apartzingan, Coatzacoalcos and Zamora, and one outbreak-district (San Nicolás), which showed an increased trend after the alarm signal (post-alarm period). See Table 4.

**Table 4:** Intensity of entomological studies. Household coverage (per 1000) before and after alarm signals and rate of change between periods in 2 outbreak and 9 non-outbreak districts with alarm signals.

	Intensity of Entomological studies (per 1000 district households)		Rate of change in households reached (per 1000 households), *Coef β (p-value)*		
	Before alarm signal	After alarm signal	Pre-alarm period (baseline)	Alarm period	Post-alarm period
**Outbreak district**	0.04	0.03	-	-	-
CD APODACA	0.01	0.01	-0.0002	-0.0001	-0.0001
SAN NICOLAS	0.06	0.05	-0.005*	0.072*	0.005*
**Non-outbreak district**	0.19	0.17	-	-	-
VERACRUZ	0.16	0.15	0.002	0.003	-0.005
MAZATLAN	0.24	0.25	0.001	0.009	-0.002
CULIACAN	0.19	0.15	0.007*	-0.110*	-0.008*
APATZINGAN	0.33	0.32	-0.002	0.022	0.002
COATZACOALCOS	0.28	0.19	-0.005*	0.001	0.003
CARDENAS	0.29	0.24	0.014*	-0.258*	-0.012
IGUALA	0.48	0.39	0.018*	-0.293*	-0.021*
MONTERREY	0.08	0.10	0.002*	0.004	-0.002*
ZAMORA	0.04	0.05	0.001	-0.032	0.002

* Significant (p-value<0.01)

The timeliness of the response activity “entomological studies” (S1 and S2 Figs) revealed late (or emergency) responses in both outbreak districts with alarm signals while in six out of nine non-outbreak districts with alarm signals, there was an increase of entomological studies as initial response, followed by more extensive routine activities. In the other districts, however, there was an initial response followed by routine entomological studies.

### Household spraying around a case (perifocal spraying)

Indoor space spraying around a case household is aligned with the WHO standards; Hudson X-Pert equipment is utilized to spray the block of houses but at least four houses around the case household [10]. The same pattern as above can be observed in relation to perifocal spraying of houses around a suspected dengue case. There was no or very little perifocal spraying in outbreak districts with alarm signals but it was quite frequent in the non-outbreak districts with alarm signals, often enhanced after an outbreak alarm (Table 5).

**Table 5:** Intensity of indoor spraying around case households (perifocal spraying). Percent of households treated before and after alarm signals and rate of change between periods in 2 outbreak and 9 non-outbreak districts with alarm signals.

	Intensity of spraying around case households (% of district households)		Rate of change in household coverage (% of district-level), *Coef β (p-value)*		
	Before alarm signal	After alarm signal	Pre-alarm period (baseline)	Alarm period	Post-alarm period
**Outbreak district**	0.04	0.01	-	-	-
CD APODACA	0.04	0.01	No or minimal control	No or minimal control	No or minimal control
SAN NICOLAS	0.04	0.01	-0.006*	0.035	0.008*
**Non-outbreak district**	0.09	0.07	-	-	-
VERACRUZ	0.08	0.11	-0.002*	-0.020	0.009*
MAZATLAN	0.06	0.04	-0.003	0.055	-0.006*
CULIACAN	0.06	0.03	0.003*	-0.029	-0.006*
APATZINGAN	0.55	0.52	-0.015	0.092	0.023
COATZACOALCOS	0.17	0.16	0.003	0.111	-0.016*
CARDENAS	0.24	0.14	0.010	-0.125	-0.020*
IGUALA	0.30	0.34	-0.007	0.369	-0.012
MONTERREY	0.01	0.001	-0.002*	0.010*	0.002*
ZAMORA	0.51	0.28	0.003	-0.284	-0.002

* Significant (p-value<0.01)

Regarding the timeliness of the response (S3 and S4 Figs), no initial response could be observed in outbreak districts with alarm signals. In contrast, five of the nine non-outbreak districts with alarm signals instantly increased the perifocal spraying after receiving the alarm signal (initial response) and then enhanced the response activities (early response). In the other four non-outbreak districts with alarm signals, initial responses continued with routine activities (definitions of initial and early response in Table 1). This pattern was confirmed by findings from the segmented time-series analysis (Table 5). The intensity of house spraying around cases was low in the outbreak districts with alarm signals and this continued to show no or minimal rates of change before and after the alarm signal, with the exception of one non-outbreak district with alarm signals (San Nicolás). Perifocal spraying in non-outbreak districts with alarm signals varied considerably among districts, and this was reflected in the trend at baseline; at the time of the alarm signal and during the period after the alarm, when most districts retained low activities.

### Indoor space spraying

Results from analyzing the indoor space spraying indicator revealed that response activities–indoor residual spraying–in outbreak districts with alarm signals were only carried out once the outbreak had begun (Table 6). In non-outbreak districts with alarm signals (with the exception of Cardenas and Monterrey districts), there were already considerable ongoing indoor spraying activities during the pre-alarm period, with a general increase of indoor spraying during the alarm period. However, the overall intensity of indoor spraying was statistically similar in both outbreak and non-outbreak groups (see Table 2).

**Table 6:** Intensity of indoor spraying before and after the alarm signal across two outbreak-districts and nine non-outbreak districts with alarm signals with the rate of change (from before to after alarm period).

	Intensity of indoor spraying (% of District Households)		Rate of change in % of households treated (% at district-level), *Coef β (p-value)*		
	Before alarm signal	After alarm signal	Pre-alarm period (baseline)	Alarm period	Post-alarm period
**Outbreak district**	0.01	0.06	-	-	-
CD APODACA	0.01	0.02	0.0001	0.012	0.0001
SAN NICOLAS	0.02	0.11	0.0001	0.091*	-0.001
**Non-outbreak district**	0.29	0.59	-	-	-
VERACRUZ-	0.62	0.47	0.023	-0.086	-0.056*
MAZATLAN-	0.02	0.03	0.002*	0.002	-0.003*
CULIACAN	0.02	0.05	0.001*	0.042*	-0.003*
APATZINGAN	1.25	3.79	0.062	2.256*	-0.109
COATZACOALCOS	0.01	0.01	0.0001	0.001	-0.001
CARDENAS	0.06	0.01	-0.009*	0.086*	0.009*
IGUALA	0.77	1.22	0.105*	-1.015*	-0.104*
MONTERREY	0.02	0.08	-0.002	0.030	0.003
ZAMORA	0.44	0.66	0.028*	0.002	-0.043*

* Significant (p-value<0.01)

Concerning the timeliness of the response in the outbreak districts with alarm signals (S5 and S6 Figs), the increase of indoor spraying occurred long after the alarm signal and only when the case numbers increased. In five out of nine non-outbreak districts with alarm signals, initial response activities started after the alarm signal, followed by early response activities. This is also illustrated in Table 6 revealing a general low coverage of indoor spraying activities (since this is a resource-intensive activity), but they were significantly higher in four out of nine non-outbreak districts with alarm signals. Unlike the case in the other three response activities, most of the districts in both the outbreak and non-outbreak groups demonstrated increasing trends of indoor spraying from baseline to the time when the alarm signal turned positive.

### Space spraying (fogging)

Despite some inconsistencies in measuring the “fogging (space spraying)” during the data collection process, which hinders further quantitative assessment, a qualitative analysis is outlined instead. For the space spraying, the covered area indicator (Km2) has been used per epidemiological week. This indicator shows a slightly different pattern compared to the other vector control components. There was an increase in activities during the alarm periods in outbreak districts with alarm signals, while, in non-outbreak districts with alarm signal the increase is minimal (S7 and S8 Figs). Culiacan, a non-outbreak district with alarm signals, presents a decrease of space spraying (fogging) just after the alarm signal is postive but the other non-outbreak districts with alarm signals show an increase of fogging which, however, is similar to the increase experienced in outbreak districts with alarm signals.

In two outbreak districts with alarm signals (San Nicolás de los Garza and Ciudad Apodaca), there was an increase in fogging activities after the alarm but this is intermittent and decreased after a few weeks to almost zero compared to the continuous fogging in non-outbreak districts with alarm signals during most of the year.

## Discussion

The EWARS, developed by WHO-TDR and partner countries, has shown in previous studies adequate prediction performance across variant settings and adapted to be operated by skilled and unskilled users, which can have significant operational implications [5,7]. However, the impact measurement of the EWARS in endemic areas remains as a crucial piece of assessment in this overall early warning and response process.

Based on experience and historical observations from the Mexican National Surveillance and Vector Control Program, it can be argued that differences between the districts-groups (outbreak- and non-outbreak districts) can be explained by the type and timeliness of the response to the alarms. This study applied a quantitative approach to examine and compare key outbreak response activities and their impact on the outbreak prevention in districts with alarms followed by outbreaks (“outbreak districts with alarm signals”), and in districts which did not have outbreaks after the alarms (“non-outbreak districts with alarm signals”). Two out of 11 districts with outbreak alarms presented outbreaks and nine had no outbreak after the alarm. The study confirmed the hypothesis that more intense vector control activities (larval control, entomological studies including additional larval control, and perifocal spraying around households of cases), were apparent in non-outbreak districts with alarm signals compared to the outbreak districts with alarm signals. Only the ‘indoor space spraying’ was in all districts reduced after the alarm in favour of other vector control measures.

It is worth noting that in routine vector control programs, the coverage of the whole district population is generally low because the vector control services focus their efforts mainly on high transmission areas (“hot spots”) where they do house-to-house visits for vector control activities. This is particularly obvious in large cities like Monterrey (with more than 1.2 million population). The use of the intensity of response in terms of proportion of houses reached or served out of all houses in the district (rather than houses in the target area as this number is unknown) is viewed as a pragramtic and scientifically feasible approach of assessing differences observed in our outbreak- and non-outbreak groups.

Delayed or non-effective response activities measured by the timing (initial, early or late response) can additionally be useful indicators for explaining the impact of the EWARS on outbreak response. Nevertheless, continuous routine activities were maintained throughout the year in the non-outbreak districts with alarm signals following the alarm signals. Larval control measure (weekly number of houses visited per week), entomological studies (number of investigations plus interventions per week) and focal spraying (number of dengue cases with focal spraying) as well as additional activities as initial or early response were observed. In contrast, in outbreak districts with alarm signals late or emergency responses were observed. The indoor space spraying (number of houses sprayed per week) was routinely practiced prior to alarm signals, but was apparently more intermittent within outbreak districts with alarm signals where only late or emergency responses were observed (compared to initial and early response in non-outbreak districts with alarm signals).

The segmented time series analysis showed plausible trends and patterns between and within the two district-groups confirming the hypothesis of our study. The baseline (routine) vector management of any of the vector control indicators appears to be important for defining the outbreak profile of the endemic area–since districts with poor baseline vector control activities are more likely to have disease outbreaks than those without. While vector control follows a routine schedule at national level, the application of routine vector control varies across districts and tends to be particularly weak when districts fail to respond to early outbreak warning. Furthermore, as shown in our analysis, the immediate response action at the week of receiving an outbreak alarm plays a crucial role in reducing the probability of having disease outbreaks. This can assist district managers to recognize the importance of a scaled response. For instance, in the two outbreak districts with alarm signals, the larval control response was delayed (emergency response) until dengue case numbers had already crossed the outbreak threshold. In the non-outbreak districts, however, five out of nine districts had started with initial response after the first alarm followed by early response activities (when the alarm continued), two had started with initial response activities but then continued with routine larval control and, two had started with early response in addition to routine activities.

The outdoor space spraying (fogging) is the only component with a different pattern and seems not to have a significant correlation with the occurrence of the outbreak. This may be due to the weakness of space fogging as an effective component to stop transmission as declared by some literature reviews [11] or potentially due to operational failures (the fogging did not reach the necessary coverage or frequency and timing were incorrect).

While the number of outbreak districts is small (two districts), which is considered a limitation in this study, this design would still reflect a real life scenario at country-level and speak to the plausibility of the approach and its findings, which is important for operational aspects. Some confounding variables that could not be taken into account in the analysis may further impact on the conclusion, such as the level of endemicity in districts and the periodicity of the transmission cycles or the possible alternation of predominant dengue serotypes. Nevertheless, findings from a robust segmented time series analysis suggest that, continuous routine vector control as enforced by additional response efforts after outbreak alarms, will likely mitigate dengue outbreaks and reduce unwanted negative consequences for health system and families. This conclusion has been reconfirmed by the mix-effect linear regression and GEE analysis of the association between the intensity of routine vector control plus enhanced control activities after an outbreak alarm.

This article neither attempts to evaluate the adherence of health district managers to the early warning and response system nor assessing the effectiveness of EWARS in terms of mitigating or averting outbreaks, which typically requires robust randomized controlled trials and accounts for additional factors related to resource availabilities and managerial aspects. However, it plausibly demonstrates important operational scenarios when succeeding or failing alarm signals generated by EWARS at national and local (district) levels. Failing timely response to alarms signals generated by EWARS showed to negatively impact the disease outbreak control process. On the other hand, districts with adequate and timely response triggered by alarm signals demonstrated successful records of outbreak prevention. Findings from this study warrant further investigation into the effectiveness and cost-effectiveness of EWARS using more robust designs. Subsequent evaluations could further include the availability of resources at the district level, since insufficient resources can naturally affect the timeliness and coverage of actions and their consequences.

## Supporting information

S1 Fig Timelines of the entomological studies as vector control and responses activities. Illustration of initial (1), early (2) and emergency/late (3) responses as practiced in outbreak districts based on the prediction generated from the EWARS.
(TIFF)

S2 Fig Timelines of the entomological studies as vector control and responses activities. Illustration of initial (1), early (2) and emergency/late (3) responses as practiced in non-outbreak districts based on the prediction generated from the EWARS.
(TIFF)

S3 Fig Timelines of the perifocal spraying as vector control and responses activities. Illustration of initial (1), early (2) and emergency/late (3) responses as practiced in outbreak districts based on the prediction generated from the EWARS.
(TIFF)

S4 Fig Timelines of the perifocal spraying as vector control and responses activities. Illustration of initial (1), early (2) and emergency/late (3) responses as practiced in non-outbreak districts based on the prediction generated from the EWARS.
(TIFF)

S5 Fig Timelines of the indoor spraying as vector control and responses activities. Illustration of initial (1), early (2) and emergency/late (3) responses as practiced in outbreak districts based on the prediction generated from the EWARS.
(TIFF)

S6 Fig Timelines of the indoor spraying as vector control and responses activities. Illustration of initial (1), early (2) and emergency/late (3) responses as practiced in non-outbreak districts based on the prediction generated from the EWARS.
(TIFF)

S7 Fig Timelines of the fogging as vector control and responses activities. Illustration of initial (1), early (2) and emergency/late (3) responses as practiced in outbreak districts based on the prediction generated from the EWARS.
(TIFF)

S8 Fig Timelines of the fogging as vector control and responses activities. Illustration of initial (1), early (2) and emergency/late (3) responses as practiced in non-outbreak districts based on the prediction generated from the EWARS.
(TIFF)
