# Supplementary figures and images for: Validation of the Early Warning and Response System (EWARS) for dengue outbreaks: Evidence from the national vector control program in Mexico
Source: PLoS Negl Trop Dis. 2021 Dec 16;15(12):e0009261. doi: 10.1371/journal.pntd.0009261 (PMC8717980; doi:10.1371/journal.pntd.0009261)

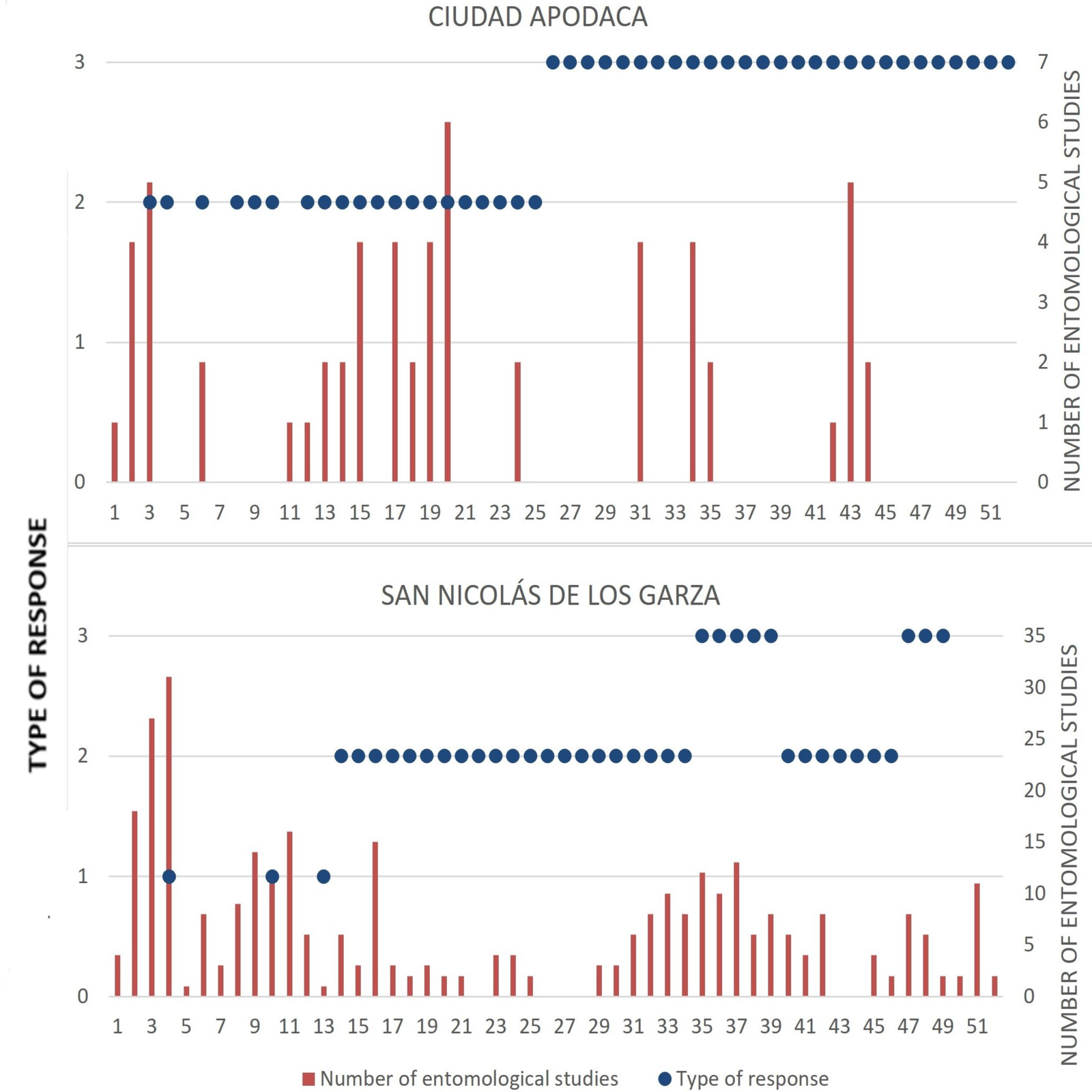

Supplement: S1 Fig — Illustration of initial (1), early (2) and emergency/late (3) responses as practiced in outbreak districts based on the prediction generated from the EWARS. (TIFF) [file pntd.0009261.s001.tiff]

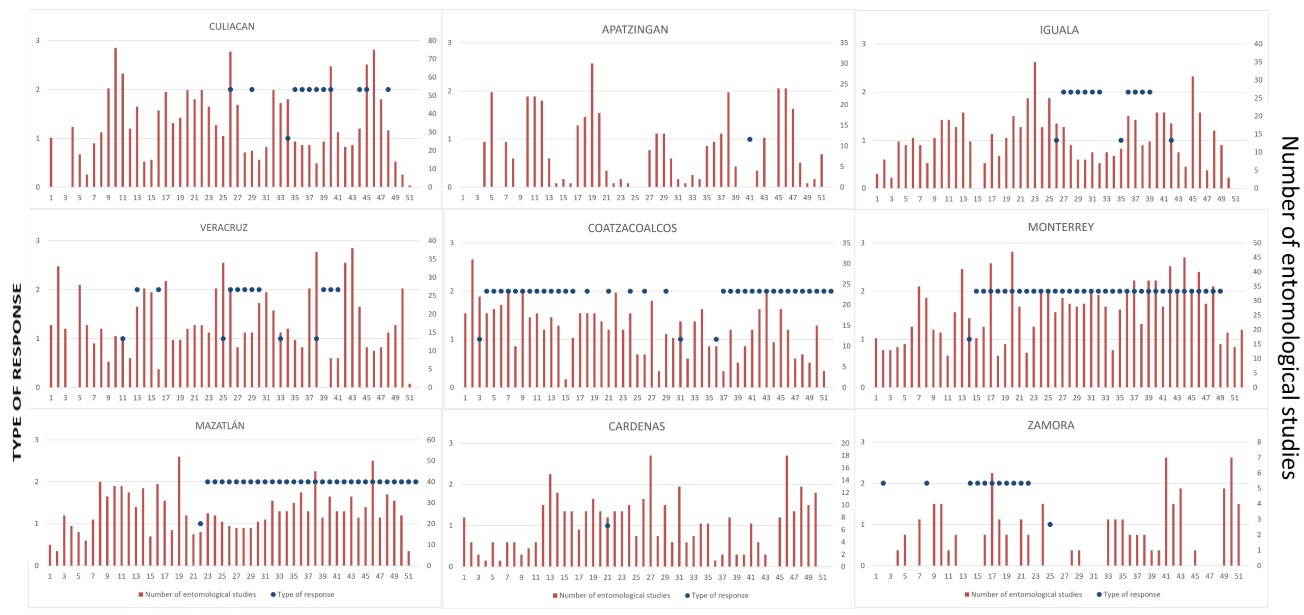

Supplement: S2 Fig — Illustration of initial (1), early (2) and emergency/late (3) responses as practiced in non-outbreak districts based on the prediction generated from the EWARS. (TIFF) [file pntd.0009261.s002.tiff]

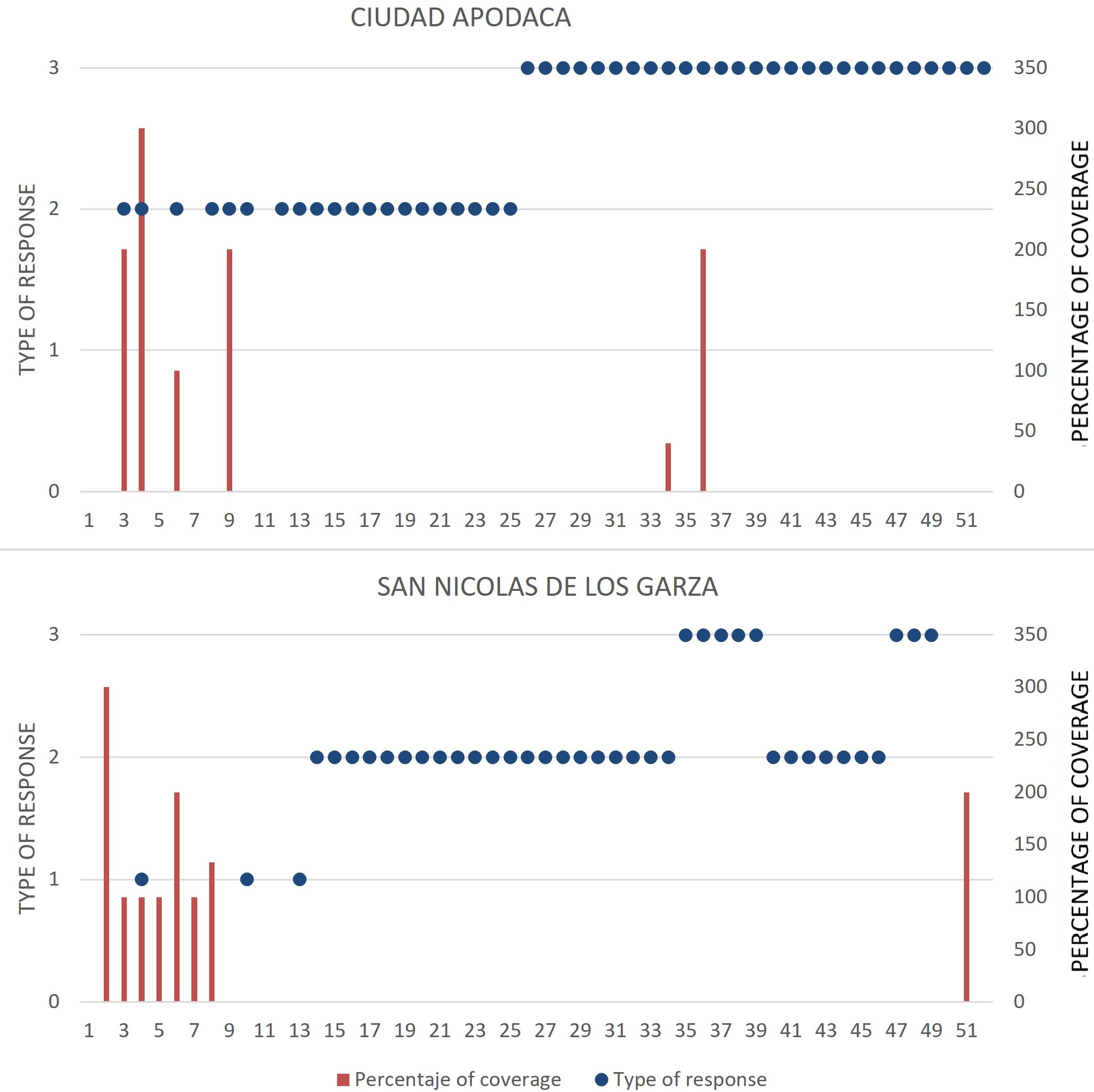

Supplement: S3 Fig — Illustration of initial (1), early (2) and emergency/late (3) responses as practiced in outbreak districts based on the prediction generated from the EWARS. (TIFF) [file pntd.0009261.s003.tiff]

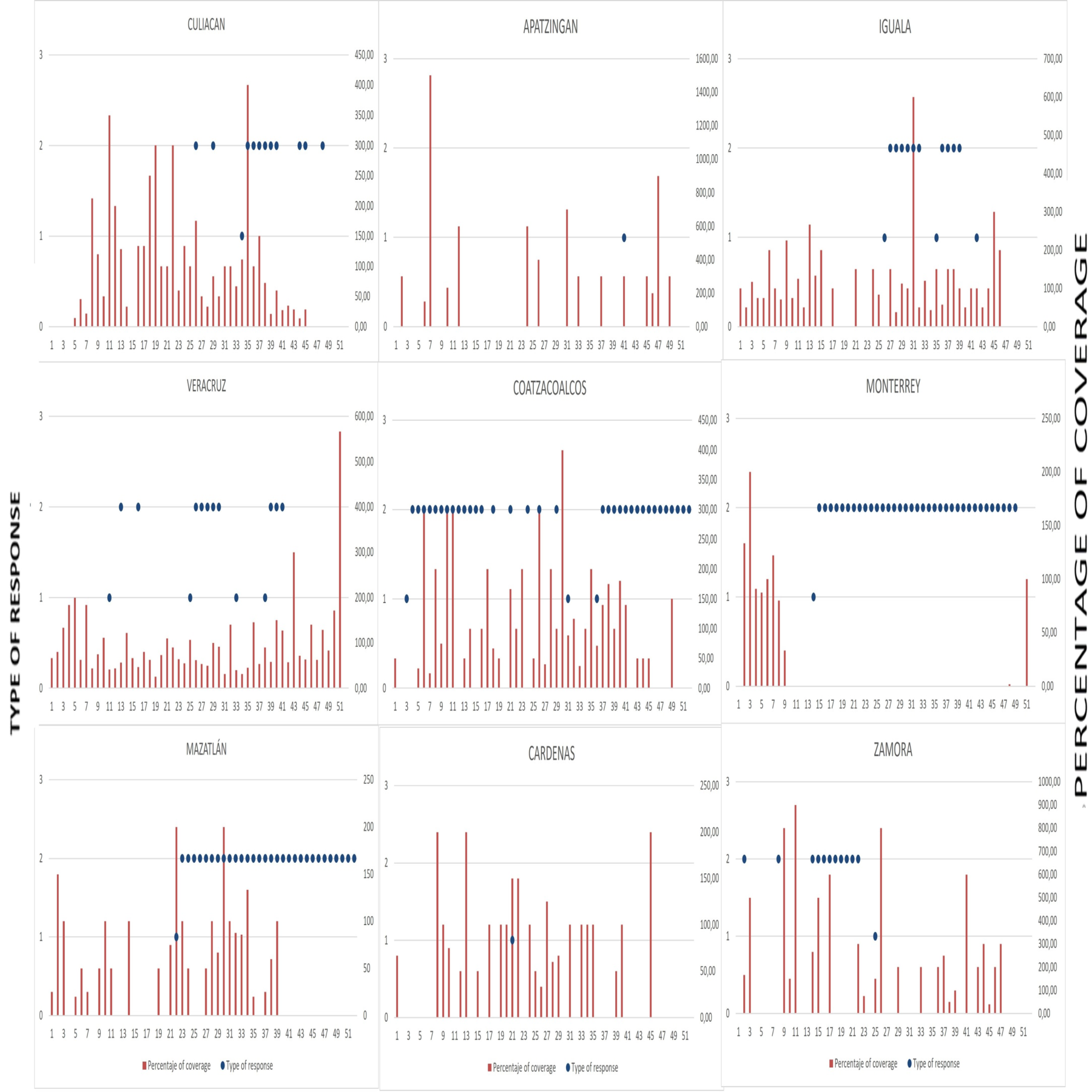

Supplement: S4 Fig — Illustration of initial (1), early (2) and emergency/late (3) responses as practiced in non-outbreak districts based on the prediction generated from the EWARS. (TIFF) [file pntd.0009261.s004.tiff]

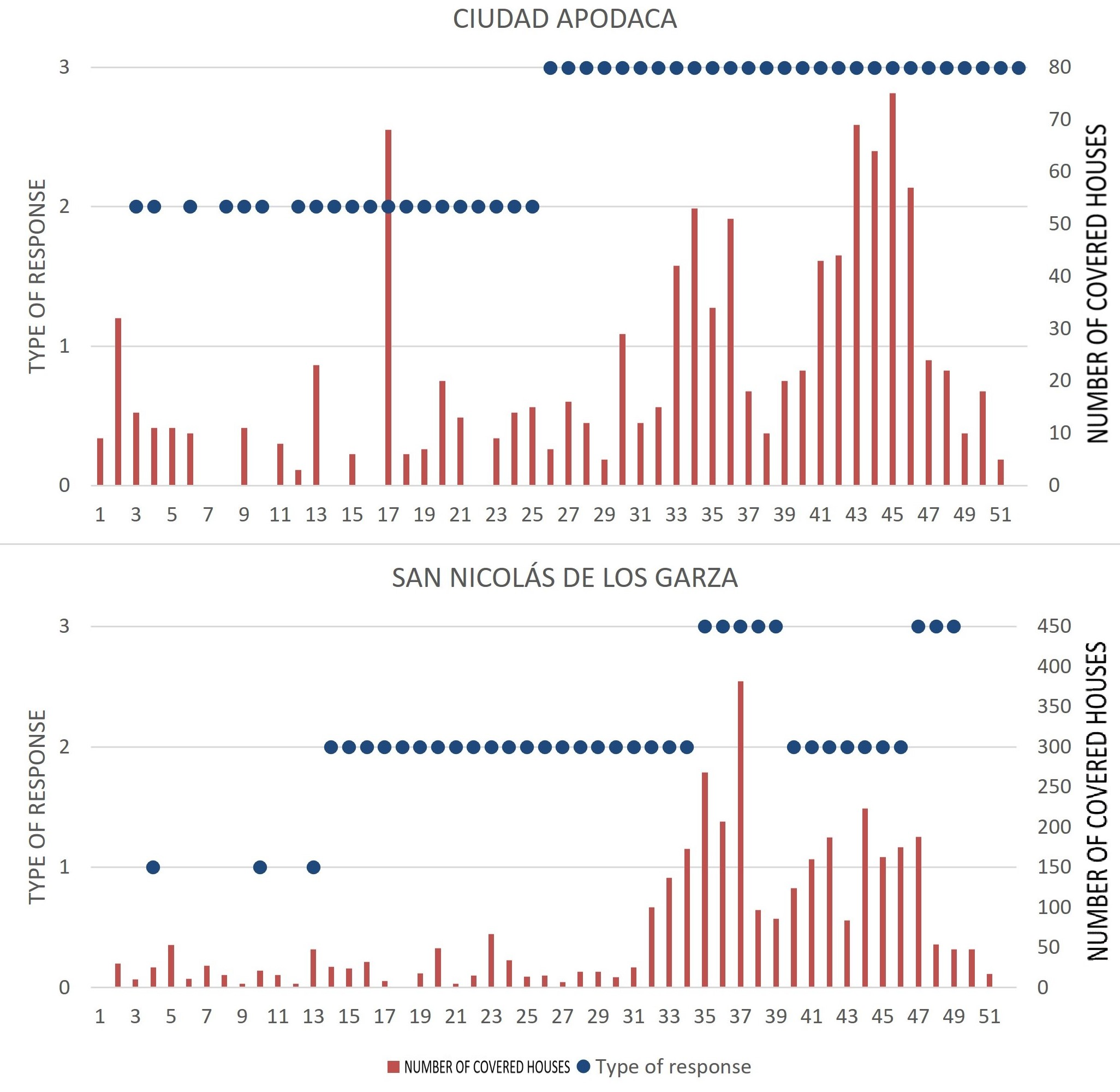

Supplement: S5 Fig — Illustration of initial (1), early (2) and emergency/late (3) responses as practiced in outbreak districts based on the prediction generated from the EWARS. (TIFF) [file pntd.0009261.s005.tiff]

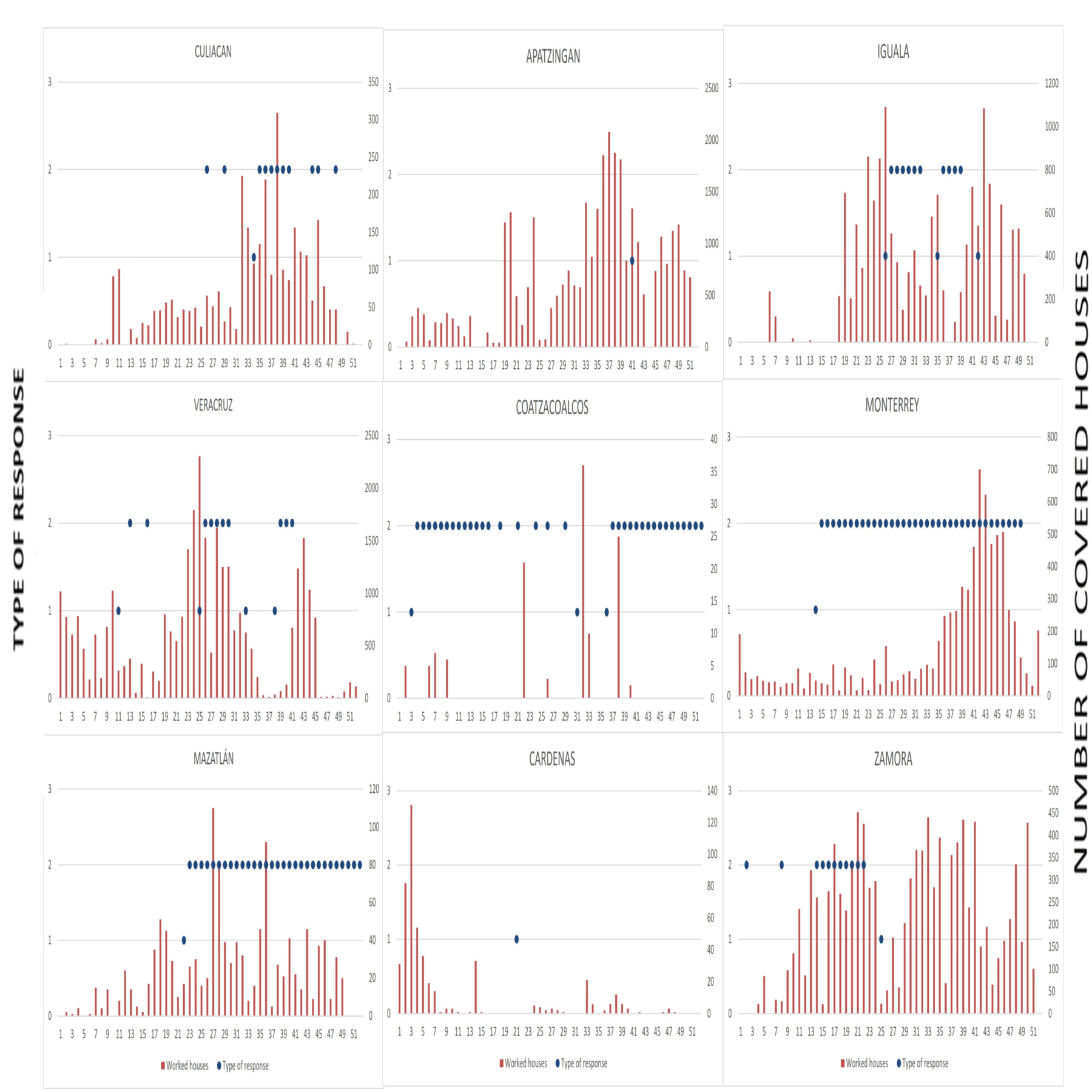

Supplement: S6 Fig — Illustration of initial (1), early (2) and emergency/late (3) responses as practiced in non-outbreak districts based on the prediction generated from the EWARS. (TIFF) [file pntd.0009261.s006.tiff]

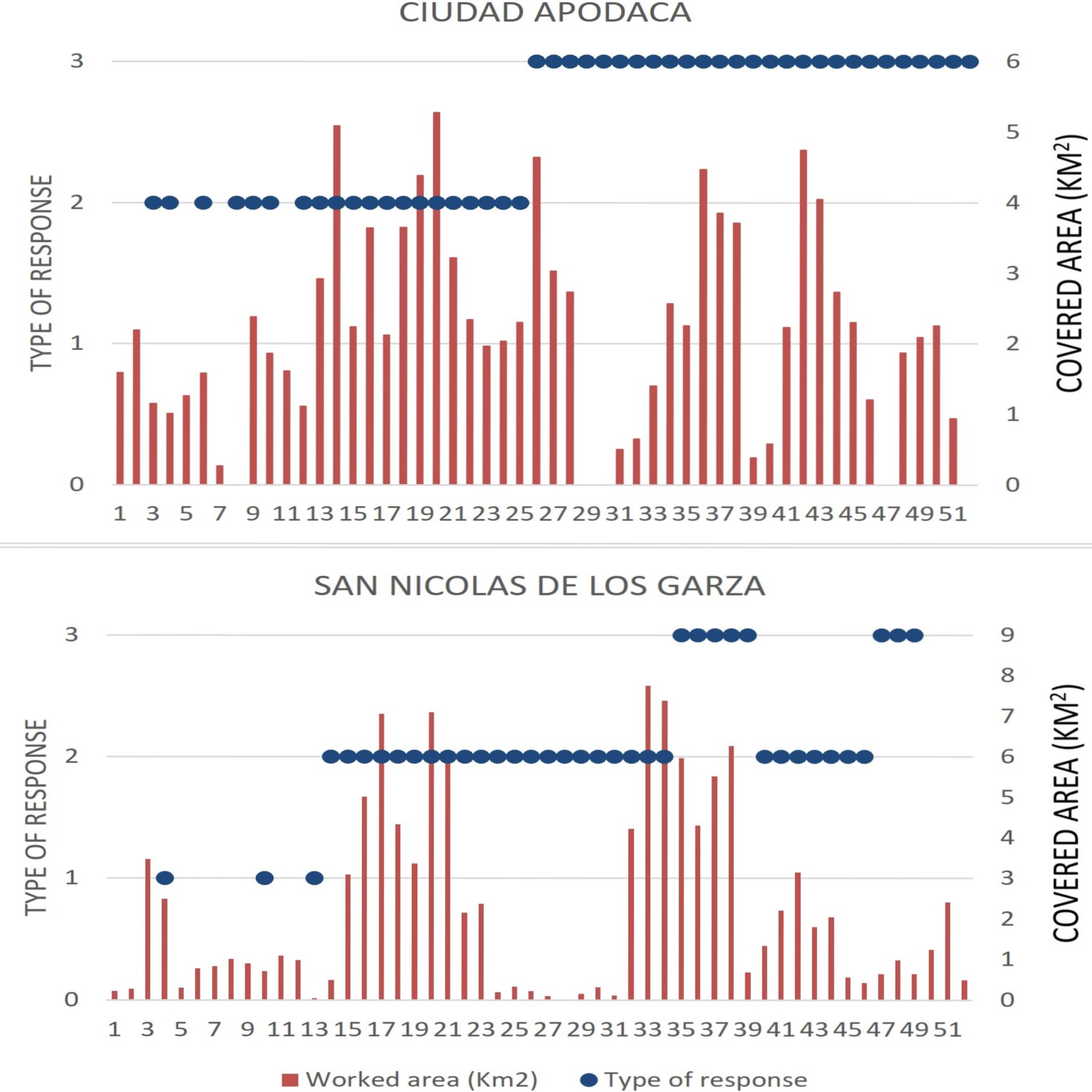

Supplement: S7 Fig — Illustration of initial (1), early (2) and emergency/late (3) responses as practiced in outbreak districts based on the prediction generated from the EWARS. (TIFF) [file pntd.0009261.s007.tiff]

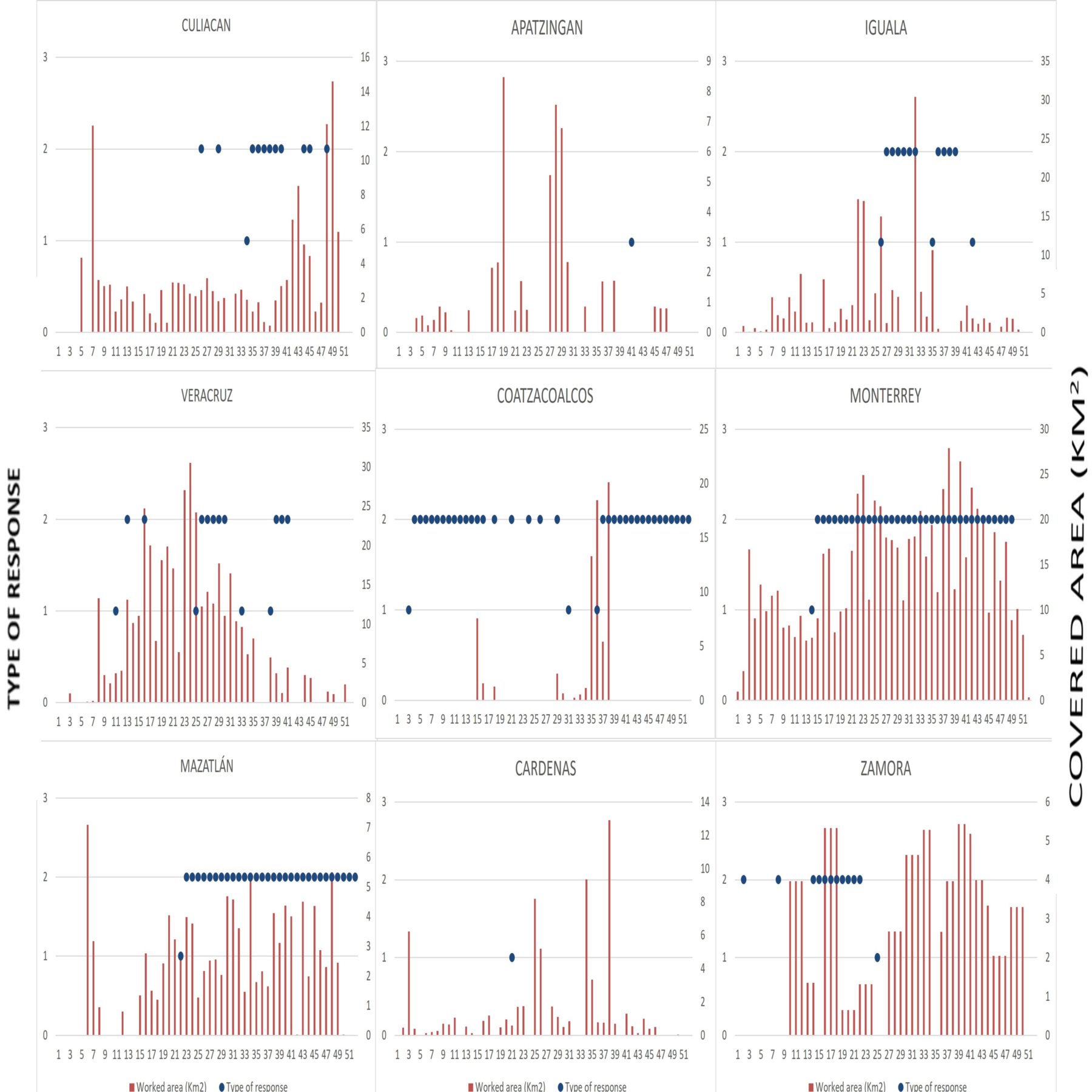

Supplement: S8 Fig — Illustration of initial (1), early (2) and emergency/late (3) responses as practiced in non-outbreak districts based on the prediction generated from the EWARS. (TIFF) [file pntd.0009261.s008.tiff]
